# Endovascular Treatment of Complex Intracranial Aneurysms With LEO+ Stents: The LEO II Cohort Study

**DOI:** 10.3389/fneur.2022.848683

**Published:** 2022-06-28

**Authors:** Omer F. Eker, Olivier Levrier, Emmanuel Houdart, Marianne Bonja, Denis Herbreteau, Alain Bonafé, Hubert Desal, the LEO+ II Investigators

**Affiliations:** ^1^Department of Neuroradiology, Hôpital Pierre Wertheimer, Hospices Civils de Lyon, Lyon, France; ^2^Department of Interventional Neuroradiology, Polyclinique Clairval, Marseille, France; ^3^Department of Neuroradiology, Hôpital Lariboisière, Paris, France; ^4^Department of Clinical Affairs, Balt Extrusion, Montmorency, France; ^5^Department of Radio-Diagnostic and Medical Imaging, Centre Hospitalier Régional et Universitaire Bretonneau, Tours, France; ^6^Department of Neuroradiology, Hôpital Gui-de-Chauliac, Montpellier, France; ^7^Department of Diagnostic and Interventional Neuroradiology, Hôpital Guillaume et René Laënnec, Nantes, France

**Keywords:** intracranial aneurysm, wide neck aneurysm, stent-assisted coil embolization, efficacy, safety, LEO+ stent

## Abstract

**Introduction:**

Stent-assisted coiling is an established treatment option for intracranial aneurysms, particularly, wide neck aneurysms with complex anatomy. The purpose of the present study was to assess the safety and efficacy of LEO+ stents in the treatment of intracranial aneurysms.

**Materials and Methods:**

A prospective, observational, multicenter study including 12 centers was performed on patients with ruptured, unruptured, and recanalized intracranial aneurysms treated with LEO+ stents. The primary efficacy endpoint was the rate of complete aneurysmal occlusion at 18 months post-procedure, and the primary safety endpoint was the morbidity and mortality at 18 months post-procedure.

**Results:**

From March 2015 to June 2017, 176 patients were enrolled (mean age of 54.8 ± 11.5 years; 65.9% women). The aneurysms were located mainly in the anterior communicating artery (29.2%) and the middle cerebral artery (28.6%). They were mostly saccular (94%), with a mean dome size of 5.3 ± 2.6 mm and a mean aspect ratio of 1.2 ± 1.0. In total, 3% of the aneurysms were treated in the acute phase of rupture. Complete/nearly complete aneurysmal occlusion and major recanalization were observed in 89% (*n* = 146/164) and 1.8% (*n* = 3/164) of patients at 18-month follow-up, respectively. The LEO+ related mortality rate was 0.6% (*n* = 1/170), the morbidity rate was 4.1% (*n* = 7/170), and these patients were mRS 0–2 at 18 months.

**Conclusion:**

Our results reflecting the use of LEO+ stents in real-world conditions confirm the efficacy and safety of LEO+ stents in the management of complex intracranial aneurysms.

## Introduction

Intracranial aneurysm (IA) is the leading cause of hemorrhagic stroke in the younger population. Numerous factors increasing the risk of IA rupture have been reported, including a size >5 mm and location in the posterior circulation (1). Many studies have reported the benefits of primary prevention treatment of unruptured IAs (2–10). Nevertheless, two factors still limit the effectiveness of endovascular treatment (EVT): a neck size ≥4 mm and an aspect ratio <1.5 (10, 11). When present, these factors define complex aneurysms (i.e., those with unfavorable anatomical configurations). In this context, the use of intracranial stents has been proposed to treat complex aneurysms (12). Stent-assisted coiling avoids the need for transient luminal occlusion with balloon remodeling. In addition, the stent creates a mechanical scaffold that prevents coil protrusion into the parent artery and promotes endothelialization at the aneurysm neck. Therefore, this method results in a significantly lower rate of angiographic recurrence than either coiling alone or coiling with balloon remodeling (13). LEO+ stents have been proposed for the treatment of complex ruptured or unruptured IAs for which simple EVT (i.e., coiling alone or balloon-assisted coiling) is difficult to perform. The present observational study was performed to assess the morbidity, mortality, and efficacy of LEO+ stents in EVT for IA and the associated clinical practice in a real-world setting.

## Materials and Methods

### Study Design

A prospective, observational, multicenter study including 12 French centers was conducted between March 2015 and January 2019. The aim of the registry study was to assess the morbidity, mortality, and efficacy of LEO+ stents (i.e., LEO+ and LEO+ baby, BALT Extrusion, Montmorency, France) in EVT for complex IAs (ClinicalTrials.gov identifier NCT03504436). The study was conducted in accordance with the ethical principles of the Declaration of Helsinki, in compliance with the approved protocol, the guidelines for good clinical practice (NF EN ISO 14155) and the rules governing medical devices, and also local regulations. Regulatory authorizations were obtained before any inclusion of participants in the study.

### Inclusion/Exclusion Criteria

Patients with IAs treated with one or more LEO+ stents who provided informed consent to participate were included in this study. Ruptured IAs were not excluded if they could not be treated without stenting in the acute phase. Because this study evaluated LEO+ device use in a real-world setting, no other eligibility criteria were defined.

### Endpoints

The primary efficacy endpoint was the rate of complete aneurysmal occlusion at 18 months post-procedure, evaluated angiographically. The occlusion was classified as complete occlusion, residual neck, or residual aneurysm according to the Montreal grading scale (14). A residual aneurysm was considered to be a treatment failure. Between the EVT and the 18-month visit, the evolution of the occlusion was classified as either stable, progressive thrombosis (i.e., improvement according to the Montreal grading scale), or recanalization (i.e., worsening according to the Montreal grading scale). The recanalization was classified as minor if no retreatment was needed and as major if re-treatment was required.

The primary safety endpoint was the morbidity and mortality rates associated with LEO+ stents and/or the procedure at 18 months post-EVT. The degree of neurological disability was assessed with the modified Rankin scale (mRS) score at a minimum of 90 days post-event. Favorable and unfavorable outcomes were defined by mRS scores of 0–2 and >2, respectively.

In addition, data associated with the patient's demographics; medical history; clinical outcomes at discharge and the 1-, 3-, and 18-month follow-ups; the aneurysm status, morphology, location and size; the antiplatelet/anticoagulant regimen used in the preparation of and post-EVT; and the details of LEO+ stent delivery practices were collected and evaluated as secondary endpoints.

An independent core laboratory assessed all the angiographic images and the efficacy endpoint. The safety endpoint was assessed by a clinical event committee, which conducted an independent analysis of the safety data.

### Statistical Analysis

Because the study was not controlled, the calculation of the number of patients required was based on the precision of the primary endpoints according to the literature (15). Because 10% of patients were non-evaluable, the final sample size was 176 patients. Descriptive statistics was used to present the results. Quantitative variables are expressed as the number of observations, mean, SD, median, 1st and 3rd quartiles, minimum and maximum, and 95% CIs. Qualitative variables are summarized as counts and percentages. The analyses were performed in the following populations: (1) patients in whom a LEO+ stent was implanted or attempted to be implanted, even without success (i.e., full analysis set, FAS); (2) evaluable patients with an implanted LEO+ stent (i.e., evaluable analysis set, EAS); and 3) evaluable patients with an implanted LEO+ and an angiographic efficacy assessment at 18 months (i.e., efficacy evaluable analysis set, EEAS). The efficacy endpoint was assessed in the EEAS, whereas the safety endpoint was assessed in the FAS. Patients who died or were lost to follow-up were included in the primary efficacy analysis as having a residual aneurysm at 18 months (i.e., treatment failure).

## Results

### Study Populations

**Figure 1:**
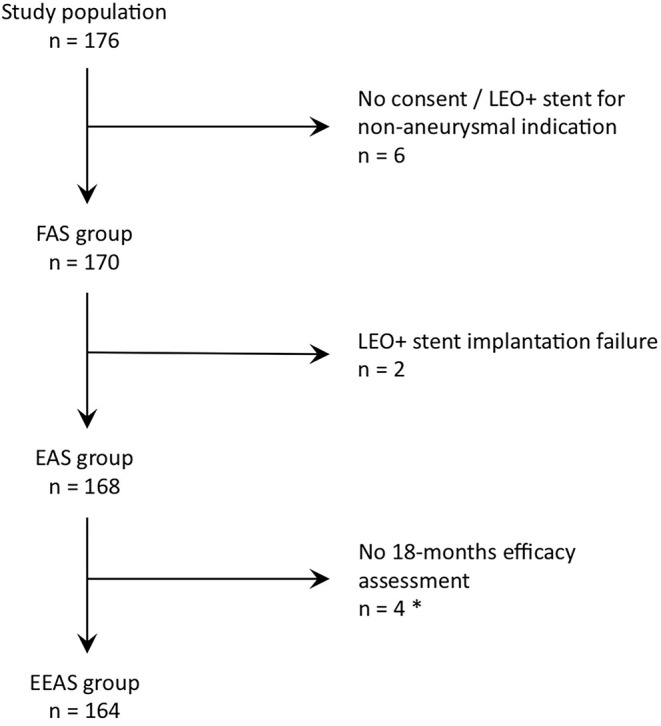
FAS, Full analysis set; EAS, Evaluable analysis set; EEAS, Efficacy evaluable set. ^*^ Four early withdrawals for non-medical reasons, one lost to follow-up patient and one deceased patient secondarily to a per procedural complication. The 2 latter patients were included in the primary efficacy endpoint analyses as a worst-case scenario (i.e., considered as residual aneurysm).

Of the 176 patients included in the study, 170 patients constituted the FAS. Consent to participate was not obtained in five patients. In one case, the LEO+ was not used for IA treatment but instead was used for an arterial dissection. Among the FAS population, in two patients, LEO+ stent implantation was attempted and failed, thus, resulting in 168 patients constituting the EAS. In the EAS group, four patients had no angiographic efficacy assessment at 18 months because of early withdrawal from the study for non-medical reasons, one patient was lost to follow-up, and one patient died after a per-procedural complication, thus, resulting in 162 patients with an angiographic assessment at the 18-month follow-up. The patient lost to follow-up and the patient who died were considered to have the worst-case scenarios in the efficacy analysis, thus, resulting in 164 patients constituting the EEAS (Figure 1). mRS assessments were available in 168 patients at inclusion and discharge, in 155 patients at 3-month follow-up and in 151 patients at the 18-month follow-up. The average follow-up time was 15.8 months.

Baseline and aneurysm characteristics are reported in Tables 1, 2, respectively. Most aneurysms included in the study were incidental, unruptured, small- or medium-sized and saccular (*n* = 158/168, 94%). Wide neck aneurysms (aspect ratio <1.5) located in the anterior communicating complex and middle cerebral artery bifurcation predominated, accounting for more than two-thirds of the aneurysms treated. Target aneurysms had been treated previously in 54/168 (32.1%) patients. Before EVT, 160/170 (94.1%) had mRS scores <2.

**Table 1:** Baseline characteristics in the FAS subgroup.

**Baseline characteristics**	***n*_FAS_ = 170**
Age, years-old (mean±SD) Female, *n* (%)	54.8 (11.5) 112 (65.9)
**Medical history**, ***n*** **(%)** Hypertension Smoking Diabetes Ruptured aneurysm Migraine Ischemic Stroke Transient ischemic attack	149 (87.6) 105 (61.8) 9 (5.3) 82 (48.2) 46 (27.1) 27 (15.9) 9 (5.3) 6 (3.5)
**Aneurysm discovery**, ***n*** **(%)**	
Incidental Symptomatic *Subarachnoid hemorrhage* *Headaches* *Oculomotricity disorder* Others*	102 (60.0) 68 (40.0) 37 (21.8) 12 (7.1) 5 (2.9) 14 (8.2)
**Pre-treatment mRS**, ***n*** **(%)**	
0 1 2 3	122 (71.8) 38 (22.4) 6 (3.5) 4 (2.4)

*Transient global amnesia, fissure syndrome, discomfort due to artery dissection, seizures, dizziness +/– vomiting, left lower limb deficiency, memory disorders +/– asthenia +/– language disorders associated with spatial disorientation, dysarthria with aphasia, ischemic stroke, and obstructive hydrocephalus.

**Table 2:** Aneurysm characteristics in the EAS subgroup.

**Aneurysm characteristics**	***n*_EAS_ = 168**
**Location**, ***n*** **(%)**	
Anterior communicating artery Middle cerebral artery Internal carotid artery Anterior cerebral artery Basilar artery Posterior communicating artery Others*	49 (29.2) 48 (28.6) 30 (17.9) 19 (11.3) 10 (6.0) 3 (1.8) 8 (4.8)
**Aneurysm status**, ***n*** **(%)**	
Previously treated aneurysm Multiple aneurysms Ruptured aneurysm treated in acute phase	54 (32.1) 49 (29.2) 5 (3.0)
**Aneurysm morphology**, ***n*** **(%)**	
Saccular Fusiform Dissecting	158 (94.0) 9 (5.4) 1 (0.6)
**Aneurysm size (mm)**	
Neck, mean±SD Dome, mean±SD Aspect ratio, (mean±SD) Maximal diameter (mm), *n* (%) <4 (very small) ≥4 and <10 (small) ≥10 and <25mm (large) ≥ 25 (giant)	4.5 ± 2.1 5.3 ± 2.6 1.2 ± 1.0 34 (20.9) 112 (68.7) 15 (9.2) 2 (1.2)

*Posterior inferior cerebellar artery, superior cerebellar artery, and posterior cerebral artery.

### Procedural Characteristics

Procedural data are reported in Table 3. Aneurysm coiling was performed with the LEO+ stent implantation in 161 (95.8%) of patients. A balloon was used for the EVT in 61 (36.3%) patients, either: (1) in a balloon-assisted coiling as first strategy in 47 (28%) patients who benefited secondarily from a LEO+ stent as bailout strategy; or (2) for angioplasty of the LEO+ stent to optimize its apposition to the arterial wall in 14 (8.3%) patients.

**Table 3:** Procedural data on the evaluable analysis set in the EAS subgroup.

**Procedural data**	
**Technique used**, ***n*** **(%)**	
Stent + coiling Stent assisted coiling as first strategy Jailing technique Microcatheterism through the stent Coiling followed by stenting as first strategy Bailout stenting* Stent + flow-diverter** Stent only***	161 (95.8) 104 (61.9) 69 (41.1) 35 (20.8) 10 (4.8) 47 (28.0) 5 (3.0) 2 (1.2)
**Number of implanted stents**, ***n*** **(%)**	
1 2	156 (92.9) 12 (7.1)
**Type of used stents**, ***n*** **(%)**	
LEO+ Baby LEO+	126 (75) 42 (25)
Number of used coils, (mean±SD)	5.7 ± 3.8
Staged procedure, *n* (%)	5 (3.0)

*LEO+ stent was used as a bailout strategy because of the parent artery compromission by coils protrusion despite a balloon assisted coiling as first strategy.

**LEO+ stent was deployed to support a flow-diverter stent.

***In one patient, the parent vessel was stented first, and the aneurysm coiling was postponed to prevent any thromboembolic event given the anatomical difficulties. In the second patient, the LEO+ stent was used to treat a recanalized aneurysm which was previously coiled. Given the small depth regarding the neck size of the recanalized pouch, it was not deemed to a complementary coiling.

The antiplatelet regimen before and after the EVT are reported in Table 4. Antiplatelet therapy to prepare patients before EVT was site dependent and used a combination of acetylsalicylic acid (166/168, 98.8%), clopidogrel (103/168, 61.3%), ticagrelor (59/168, 35.1%), or prasugrel (21/168, 12.5%). Monotherapy was used in 41/168 (24.4%) patients, and dual therapy was used in 118/168 (70.2%) patients. In 2/168 (1.2%) patients, a preparation with a triple therapy was administered. Unfortunately, the reasons for this antiplatelet regimen were not provided. In addition, 93.5% (157/168) of patients received heparin during the EVT.

**Table 4:** Antiplatelet therapy regimens.

	**Pre-procedure** ***n*****_EAS_ = 168**	**At discharge** ***n*****_EAS_ = 167**	**At 3 months** ***n*****_EAS_ = 159**	**At 18 months** ***n*****_EAS_ = 155**
No APT, *n* (%)	7 (4.2)	0	11 (6.9)	92 (59.4)
Monotherapy^a^, *n* (%)	41 (24.4)	7 (4.2)	78 (49.1.)	55 (35.5)
Bitherapy^b^, *n* (%)	118 (70.2)	160 (95.8)	70 (44.0)	8 (5.2)
Tritherapy^c^, *n* (%)	2 (1.2)	0	0	0

*APT, Antiplatelet therapy; ^a^A P2Y12 inhibitor (clopidogrel or prasugrel) in 40 (97.5%) patients and ASA in one (2.4%) patient; ^b^ASA associated with a P2Y12 inhibitor (clopidogrel, prasugrel, or ticagrelor) in; 116 (98.8%) patients, and association of two P2Y12 inhibitors (clopidogrel and prasugrel) in 2 (1.7%) patients; ^c^ASA and two P2Y12 inhibitor (clopidogrel and prasugrel)*.

### Efficacy

The efficacy results (immediately post-procedure and at 18-month follow-up) are reported in Table 5.

**Table 5:** Primary efficacy endpoint on the efficacy evaluable analysis set in the EEAS subgroup.

**Occlusion status**	**Immediate post-procedure** *n*_**EEAS**_ **= 164**	**18 months** *n*_**EEAS**_ **= 164**
Complete occlusion, *n* (%)	77 (47.0)	123 (75.0)
Residual neck, *n* (%)	28 (17.1)	23 (14.0)
Residual aneurysm, *n* (%) ^*^	59 (36.0)	18 (11.0)

*Aneurysms of 2 patients (deceased and lost to follow-up) were considered as residual aneurysms.

The complete/nearly complete occlusion rate was 64% (*n* = 105/164) immediately after the procedure. This rate increased to 89% (*n* = 146/164) at the 18-month follow-up. Residual aneurysms were observed in 36% (*n* = 59/164) of patients immediately after EVT and in only 11% (18/164) at the 18-month follow-up.

Stable occlusion (i.e., stable complete occlusion or stable residual neck between immediately post-procedure and the 18-month follow-up evaluations) was observed in 85/164 (51.8%) patients, whereas 68/164 (42%) patients showed improved occlusion status (i.e., evolving from residual neck to complete occlusion or from residual aneurysm to residual neck or complete occlusion between immediately post-procedure and the 18-month follow-up evaluations). Recanalizations were observed in 9/164 (5.5%) patients: 6/164 (3.7%) were minor recanalizations, and 3/164 (1.8%) were major re-canalizations deemed eligible for retreatment. All together, these evolutions resulted in a global increase in patients with complete/near complete occlusions of +41 (+25%) at the 18-month follow-up.

At the 18-month follow-up, the results, excluding the patients treated with flow-diverter stents in combination with LEO+ stents (*n* = 5/168, 3%) were as follows: complete occlusion in 74.8% (*n* = 119/154), residual neck in 14.5% (*n* = 23/159), and residual aneurysm in 10.7% (*n* = 17/159) of patients.

### Safety and Clinical Evolution

Procedure- and/or device-associated adverse events resulting in permanent morbidity and mortality occurred in 8/170 (4.7%) patients. These adverse events occurred either per- or immediately post-procedurally and included: a guidewire-associated vessel perforation (*n* = 1/170, 0.6%) resulting in subarachnoid hemorrhage (SAH); a guidewire-associated aneurysm perforation (*n* = 1/170, 0.6%) resulting in SAH, which was associated with intrastent thrombosis; an anterior communicating artery dissection (*n* = 1, 0.6%) leading to severe SAH and the patient's death; and ischemic stroke (*n* = 5, 2.9%).

Apart from one ischemic stroke, all the adverse events were directly related to LEO+ stents (*n* = 7/170, 4.1%). No complications occurred in patients treated with additional flow diverter stents. None of these events led to an mRS score >2.

Adverse events resulting in no morbidity occurred in 37/170 (21.8%) patients and included: severe arterial vasospasms that required intra-arterial nimodipine administration (*n* = 3/170, 1.8%); failures in stent deployment (*n* = 2/170, 1.2%); stent mispositioning (*n* = 1/170, 0.6%), intra- or extra-cranial carotid dissections without any clinical consequences (*n* = 3/170, 1.8%); aneurysm perforation (*n* = 1/170, 0.8%); aneurysm rupture during groin puncture (*n* = 1/170, 0.6%); and SAH (*n* = 1, 0.6%) or IPH (*n* = 1, 0.6%) that resolved without sequelae. In addition, 14/170 (8.2%) patients had per-procedural intrastent clotting that resolved without any clinical consequences after administration of abciximab (0.25 mg/kg as an intravenous bolus immediately followed by continuous intravenous infusion of 0.125 μg/kg/min for 12 h) in 11/170 (6.5%) patients, and 15/170 (8.8%) patients had immediate post-operative groin hematoma that was resolved by immediate compression.

Among the patients with morbidity associated with EVT, 3/151 (2.0%) patients had a worsened mRS at the 18-month follow-up. One patient (0.6%) with a giant basilar aneurysm developed a posterior fossa mass effect syndrome despite stage procedure treatment (mRS scores of 1 at inclusion and 2 at the 18-month follow-up). A second patient (0.6%) had clinical deterioration following surgery for a 2nd giant carotid aneurysm not treated with LEO+ stents (mRS scores of 1 at inclusion and 4 at the 18-month follow-up). The 3rd patient was the one who died after the anterior communicating artery dissection (mRS scores of 1 at inclusion and mRS score of 6 at discharge).

The mRS remained stable in 124/151 (82.7%) patients and improved in 24/151 (16.0%) patients. In total, at 18 months post-procedure, favorable outcomes (mRS = 0–2) were observed in 148/151 (98.0%) of the patients. Table 6 reports the mRS changes from inclusion to the 18-month follow-up.

**Table 6:** Clinical evolution.

		**Inclusion** *n =* **168**	**Discharge** *n =* **168**	**3 months** *n =* **155**	**18 months** *n =* **151**
mRS score *n* (%)	0 1 2 3 4 5 6	121 (72.0%) 37 (22.0%) 6 (3.6%) 4 (2.4%) 0 0 0	134 (79.8%) 22 (13.1%) 10 (6.0%) 1 (0.6%) 0 0 1 (0.6%)	123 (79.4%) 24 (15.5%) 6 (3.9%) 1 (0.6%) 0 0 1 (0.6%)	123 (81.5%) 21 (13.9%) 4 (2.6%) 1 (0.7%) 1 (0.7%) 0 1 (0.7%)

Regarding implant evolution, at the 3-month follow-up, one of the 164 patients (0.6%) presented asymptomatic intra-stent stenosis of less than 50% of the vessel diameter that did not require any additional EVT. In another 164 patients (0.6%), stent shortening was observed without any symptoms or consequences in aneurysm occlusion. No parent artery occlusion was observed during the follow-up period.

## Discussion

Stent-assisted coiling is considered a valuable tool to treat complex wide neck aneurysms, thus, yielding stable and durable anatomical results over time (16, 17). Numerous devices have been proposed for this purpose, including first- and second-generation stents. Because of their design, particularly, their cell size, first-generation laser cut stents remained vulnerable to coil prolapse and were technically challenging to deploy across complex anatomies with marked curves and tortuous segments. To address these limitations, second-generation nitinol braided stents, such as LEO+, were developed. These implants have better conformability to the vessel anatomy, even in the marked curves or tortuous vessels, and their small cell size prevents coil prolapse and provides improved support of the coil mass (17).

This observational study was conducted to assess the morbidity, mortality, and efficacy associated with the use of LEO+ devices in treating wide neck aneurysms with complex anatomy in a real- world setting. The characteristics of our population and the treated aneurysms were comparable with those reported previously in tests of similar second-generation implants (mainly saccular aneurysms with a mean neck size >4 mm and a mean dome size >5 mm, located on the anterior communicating, middle cerebral, and distal internal carotid artery segments) (18, 19).

Regarding the primary efficacy endpoint, we observed complete/nearly complete occlusion in 89% of patients at 18 months, and recanalization and retreatment rates of 5.6 and 1.9%, respectively. These results were consistent with findings from previous case series examining nitinol braided self-expanding intracranial stents in a similar population, which has reported 79.1–92.4% complete occlusion, and 5.1 and 0% recanalization and retreatment rates, respectively (18, 19). A recent meta-analysis has compared the results obtained with LEO and LVIS devices in 35 studies evaluating 1,426 patients. At an average radiological follow-up of 10.4 months, complete/nearly complete occlusion was obtained in 88.6 and 87.8% of cases, respectively (20). All our patients with complete occlusion were treated in a single procedure, except for patients for whom the treatment was staged with a stenting first and secondary coiling because of their giant aneurysms. More recently, the long-term efficacy of LEO+ stents over 5 years of follow-up has been reported (21). Retrospective work based on a more limited but demographically comparable population (*n* = 101) has indicated a rate of complete or near-complete occlusion of 87.2%, a value comparable to our results. However, unexpectedly, the rate of stent deployment failure in that study was as high as 15.8%, mainly, because of anatomical difficulties, severe vasospasms, and implant instability. In contrast, in our work, LEO+ stent deployment failed in only 1.2% of cases.

Interestingly, the major components of this cohort, as in previously reported cohorts on comparable devices, were the aneurysms on the distal bifurcations, thus, highlighting that a major advantage of braided nitinol stents is their improved navigability. This improvement in distal accessibility with respect to that of previous stents enables treatment of aneurysms in smaller arterial vasculature.

Regarding the safety primary endpoint, we observed comparable morbidity rates and a lower mortality rate at the 18-month follow-up in our case series, as compared with previously reported rates (0.6 vs. 2.0% and 3.3%, respectively) (18, 19). In addition, we observed only one delayed intra-stent stenosis, which was not significant (i.e., <5% reduction of the vessel diameter without any clinical consequences).

Notably, the data previously reported in the literature were based on studies with variable methodological designs, particularly regarding the definitions of the primary endpoints and the clinical events considered in the calculation of morbidity rates. Our work relied on conservative and exhaustive collection of data on the complications and clinical events. The permanent procedural morbidity rate in the LEO II study compares favorably with rates reported in the published literature. Thus, LEO+ stents can reasonably be considered to provide at least similar safety to other available stents.

## Limitations

The primary limitation of this study relates to the study design (single-arm, observational, and open-label study). Potential biases were minimized by appointing independent personnel who verified the data sources and analyzed the data. In addition, the very low rate of loss to angiographic follow-up due to early withdrawal, death, or true loss to follow-up−8/170 (4.7%) patients—is a strength of our analysis. Measurement bias was controlled by the establishment of a core laboratory of independent experts who were responsible for the centralized, impartial, and uniform review of all the angiographic images, and by the independent analysis of safety data by a clinical event committee. Finally, no platelet inhibition testing was required before the EVT, thus, preventing any conclusion from being drawn regarding the number of per-procedural intra-stent thromboses. Notably, most of these cases (14/15, 93.3%) resolved favorably after medical treatment without any clinical consequences even during the follow-up. Any potential resistance to antiplatelet therapies in these patients might have played a role in the occurrence of the thrombotic events, and therefore, cannot be ruled out. This limitation should be overcome using platelet inhibition tests before EVT for IA with intracranial stents to better understand the occurrence of the thrombotic events post-EVT.

## Conclusion

This is the largest prospective cohort study of patients with complex aneurysms treated with LEO+ stents to date. Our results reflect the use of LEO+ stents in the real-world conditions and confirm their safety and efficacy in the management of complex aneurysms with a wide neck.

## Data Availability Statement

The raw data supporting the conclusions of this article will be made available by the authors, without undue reservation.

## Ethics Statement

The studies involving human participants were reviewed and approved by the French Comité Consultatif sur le Traitement de l'Information en matière de Recherche dans le domaine de la Santé (CCTIRS; File n°13.752bis) and Commission nationale de l'informatique et des libertés (CNIL; Decision DR-2014-342) (ClinicalTrials.gov Identifier: NCT03504436). The patients/participants provided their written informed consent to participate in this study.

## Author Contributions

HD and OE contributed to the conception and design of the study. OE wrote the first draft of the manuscript and the iterate versions. All authors contributed substantially to the work described by actively participating in the study, in compliance with the protocol, and critically revising the manuscript for important intellectual content.

## Funding

This study received funding from BALT EXTRUSION.

## Conflict of Interest

This study received funding from BALT EXTRUSION SAS. The funder had the following involvement with the study: sponsor. For activities unrelated to the conduct and publication of the study: OE and OL report fees to institution/association from BALT EXTRUSION SAS during the conduct of the study: contract research. AB reports personal fees from BALT EXTRUSION SAS during the conduct of the study: contract consulting. For activities related to the conduct and publication of the study: OE, OL, AB, EH, DH, and HD report personal fees from BALT EXTRUSION SAS during the conduct of the study: either contract research or contract consulting. MB reports being an employee of BALT EXTRUSION SAS as Clinical Science Manager.

## Publisher's Note

All claims expressed in this article are solely those of the authors and do not necessarily represent those of their affiliated organizations, or those of the publisher, the editors and the reviewers. Any product that may be evaluated in this article, or claim that may be made by its manufacturer, is not guaranteed or endorsed by the publisher.
